#  Synthesis and Biological Evaluation of New Tricyclic Dihydropyridine Based Derivatives on Potassium Channels

**Published:** 2016

**Authors:** Miyase Gözde Gündüz, Yesim Kaya, Rahime Şimşek, Inci Sahin-Erdemli, Cihat Şafak

**Affiliations:** a *Department of Pharmaceutical Chemistry, Faculty of Pharmacy, Hacettepe University, 06100, Ankara-Turkey. *; b *Department of Pharmacology, Faculty of Pharmacy, Hacettepe University, 06100, Ankara-Turkey.*

**Keywords:** 1, 4-Dihydropyridine, Potassium channel, Pinacidil, Docking

## Abstract

The present study reports a microwave-assisted method for the synthesis of twelve novel tricyclic 1,4-dihydropyridine derivatives in which dimethyl-substituted cyclohexane and / or tetrahydrothiophene rings are fused to the DHP ring. The structures of the compounds were confirmed by spectral methods and elemental analysis.

The potassium channel opening effects of the compounds were determined on rat mesenteric arteries and urinary bladders. The obtained results indicated that some compounds produced mesenteric artery-selective relaxant properties and the effects of these compounds were mediated through ATP-sensitive potassium channels. The replacement of the second tetrahydrothiophene ring with dimethyl-substituted cyclohexane ring led to more active compounds.

Docking studies were carried out to understand the interactions of the compounds with the active site of potassium channel. The unsubstituted nitrogen atom on the 1,4-dihydropyridine ring and one of the sulfonyl oxygens were found to be important for the formation of hydrogen bonds to stabilize the compound in the center of the cavity. The nature and position of phenyl ring substituents were also effective on the activity of the compounds. Finally, a theoretical study was established to predict the ADME of the most active compounds.

## Introduction

Ion channels are macromolecular protein tunnels that allow the passage of charged ions through hydrophobic membranes and therefore help to establish and control the small voltage gradient that exists across the plasma membrane of all living cells (1, 2). Because of the contribution of ion channels to the pathophysiology of several human diseases, these membrane proteins are the targets of various drugs for therapeutic interventions (3).

Potassium channels are a diverse and ubiquitos family of membrane-spanning proteins that selectively conduct potassium ions across the cell membrane along its electrochemical gradient in both excitable and nonexcitable cells (4, 5). Their activity may be modulated by neurotransmitters, voltage, calcium and the signalling pathways they stimulate (6). 

Potassium channel opening is a physiological mechanism by which excitable cells exploit to maintain or restore their resting state (7). Modulation of potassium channels provide potentially useful approaches to treat pathological conditions by playing important roles in cellular signaling processes regulating neurotransmitter release, heart rate, insulin secretion, neuronal excitability, smooth muscle contraction and cell volume regulation (8-11).

Potassium channels exist as several types with multiple subtypes and are often classified according to their electrophysiological and pharmacological properties (12). 

Adenosine triphosphate (ATP)-sensitive potassium channels (K_ATP_) are one of the major classes of potassium channels located in many tissues including smooth muscle in the vasculature and the bladder (13, 14). K_ATP_ channels have critical importance in coupling energy metabolism of the cell and excitability of the plasma membrane (15). K_ATP_ channel openers lead to an efflux of potassium ions from the cell, which results in hyperpolarization, decreased Ca^2+^ influx and inhibition of contraction (16).

Calcium activated potassium ion channels (K_Ca_ channel), found in a wide range of different tissues and in virtually all multicellular organisms, open in response to the presence of calcium ions or other signaling molecules (17). The open probability of these channels is increased by the elevation of cytosolic calcium to cause membrane hyperpolarization. The activity of K_Ca_ channels is implicated in many physiological processes, including neurosecretion, smooth muscle contraction and action potential shape (4, 18). 

**Figure 1:**
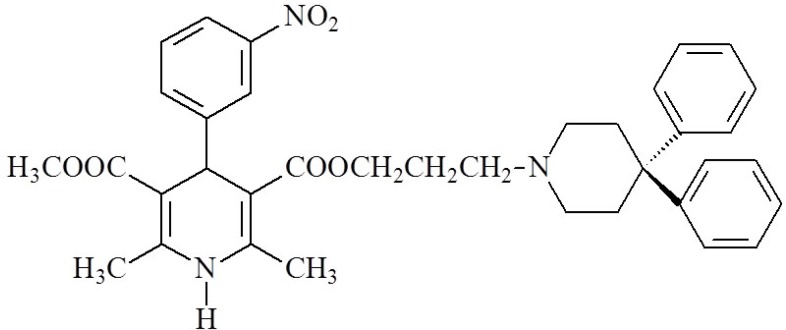
Niguldipine

1,4-Dihydropyridines (DHPs) are an important class of L-type calcium channel blockers that mainly exert their pharmacological activity by modulating Ca^2+ ^influx and are used to treat cardiovascular conditions such as hypertension and angina (19, 20). It has been reported that some 1,4-DHP derivatives that were originally developed as long-acting calcium channel blockers, such as niguldipine (Figure 1.) increased the open probability of Ca-activated potassium channels and modulated potassium current in vascular smooth muscle (21). Tricyclic dihydropyridine-based analogues, comprising a variety of heterocyclic rings fused to the dihydropyridine nucleus, were also found to be active as potassium channel openers (9, 22, 23).

Microwave (MW) irradiation as an energy source for the activation of chemical reactions has been recently introduced and gained great popularity compared to conventional reactions because of its ability to reduce reaction times, to improve yields and to simplify the work-up processes (24, 25).

Conventional reactions to obtain 1,4-DHP derivatives were also performed by applying this technique; ethanol was proved to be a much better solvent in terms of yield than the other ones including tetrahydrofuran, acetonitrile and water (26-28).

The aim of the present study is to report a rapid and convenient method based on microwave irradiation for the synthesis of twelve novel tricyclic 1,4 DHP derivatives in which dimethyl-substituted cyclohexane or tetrahydrothiophene rings are fused to the DHP ring, and to determine how the nature and position of phenyl ring substituents affect the potassium channel opening effects of these compounds on rat mesenteric arteries and urinary bladders.

## Methods and Materials

*Chemistry*

All chemicals used in this study were purchased from Aldrich and Fluka (Steinheim, Germany). All reactions were carried out in Discover Microwave Apparatus (CEM). Thin layer chromatography (TLC) was run on Merck aluminium sheets (Darmstadt, Germany), Silica gel 60 F_254_, mobile phase ethyl acetate-hexane: (1:1) and ultraviolet (UV) absorbing spots were detected by short-wavelength (254 nm) UV light (Camag UV Cabinet, Wiesloch, Germany). Melting points were determined on a Thomas Hoover Capillary Melting Point Apparatus (Philadelphia, PA, USA) and were uncorrected. ^1^H-NMR spectra were obtained in dimethyl sulfoxide (DMSO) solutions on a Varian Mercury 400, 400 MHz High Performance Digital FT-NMR Spectrometer (Palo Alto, CA, USA). Chemical shifts are reported in parts per million (ppm) relative to tetramethylsilane. The ESI-MS spectra were measured on a micromass ZQ-4000 single quadruple mass spectrometer. Elemental analyses were performed on a Leco CHNS-932 Elemental Analyzer (Philadelphia, PA, USA). 

*Synthesis of tetrahydrothiophene-3-one-1,1-dioxide:*

 Tetrahydrothiophen-3-one (0.1 mol), triethyl orthoformate (0.1 mol), p-toluensulfonic acid (0.26 mmol) and 2 mL ethanol was stirred for 20 h. The mixture was treated with anhydrous sodium acetate (4 mmol), sodium tungstate dehydrate (0.00085 mmol) and 28 mL water. 28 mL of a 35 % solution of hydrogen peroxide in water was added dropwise while keeping the reaction cooled to 30 ^°^C. After stirring overnight at room temperature, the resulting product was filtered and washed with water to achieve 3,3-diethoxytetrahydrothiephene-1,1-dione. The product was stirred in a mixture of HCl and water at 60 °C for 2 h. The mixture was extracted with dichloromethane (60 mL). The dichloromethane layer was isolated and dried with MgSO_4_, filtered and concentrated. The residue was crystallized from ethanol to provide tetrahydrothiophene-3-one-1,1-dioxide (29). 

*General procedure for the preparation of 8-(disubstituted phenyl)-2,3,4,5,6,8-hexahydrodithieno[3,2-*b*:2´,3´-*e*]pyridine-1,1,7,7-tetraoxide (Compound 1-6): *

One-pot three component mixture of 2 mmol tetrahydrothiophene-3-one-1,1-dioxide, 1 mmol appropriate disubstituted benzaldehyde and 5 mmol ammonium acetate was filled into 10 mL-microwave pressure vial and heated under microwave irradiation (power 75 W, maximum temperature 130 °C) for 5 min in 5 mL ethanol. After the reaction was completed, the reaction mixture was poured into ice-water, the obtained precipitate was filtered and crystallized from ethanol-water.

*General procedure for the preparation of 7,7-Dimethyl-9-(disubstituted phenyl)-2,3,5,6,7,9-hexahydrothieno[3,2-b]quinolin-8(4H)-one, 1,1-dioxide (Compound 7-12):*

1 mmol tetrahydrothiophene-3-one-1,1-dioxide, 4,4-dimethyl-1,3-cyclohexanedione, 1 mmol appropriate disubstituted benzaldehyde and 5 mmol ammonium acetate were filled into 10 mL-microwave pressure vial and heated under microwave irradiation (power 75 W, maximum temperature 130 °C) for 5 min in 5 mL ethanol. After the reaction was completed, the solvent (ethanol) was removed via a rotary evaporator and the crude product was then purified by column chromatography using silica gel as the solid phase and a 7:3 mixture of ethyl acetate: methanol as mobile phase.

*8-(2-chloro-5-nitrophenyl)-2,3,4,5,6,8-hexahydrodithieno[3,2-*b*:2’,3’-*e*]pyridine-1,1,7,7-tetraoxide (Compond 1)*

Yield: 82%. m.p. 248-250 ^o^C. IR (ν, cm^-1^): 3337 (N-H), 1638 (C=O), 1355, 1129 (S=O). ^1^H-NMR (*δ*, DMSO-*d*_6_): 2.18-3.51 (8H; m; H^2,3,5,6^), 4.61 (H; s; H^8^), 6.32 (H; s; NH), 7.81 (1H; d; J: 8.4 Hz; Ar-H^3^), 8.16 (1H; dd; J: 2.8 / 8.4 Hz; Ar-H^4^), 8.30 (1H; d; J: 2.8 Hz; Ar-H^6^), ESI-MS (m/z): 439.99 [M+1+Na]^+^, 438.99 [M+Na]^+ ^(100%). Anal. Calcd. for C_15_H_13_ClN_2_O_6_S_2_: C, 43.22; H, 3.14; N, 6.72; S, 15.38. Found: C, 43.23; H, 3.12; N, 6.76; S, 15.32.

*8-(2-nitro-5-chlorophenyl)-2,3,4,5,6,8-hexahydrodithieno[3,2-*b*:2´,3´-*e*]pyridine-1,1,7,7-tetraoxide (Compond 2)*

Yield: 76%. m.p. 232-234 ^o^C. IR (ν, cm^-1^): 3345 (N-H), 1632 (C=O), 1289, 1096 (S=O). ^1^H-NMR (*δ*, DMSO-*d*_6_): 2.22-3.38 (8H; m; H^2,3,5,6^), 4.52 (H; s; H^8^), 5.92 (H; s; NH), 7.45 (1H; d; J: 2.4 Hz; Ar-H^6^), 7.98 (1H; dd; J: 2.4 / 8.8 Hz; Ar-H^4^), 8.30 (1H; d; J: 8.8 Hz; Ar-H^3^), ESI-MS (m/z): 439.99 [M+1+Na]^+^, 438.99 [M+Na]^+ ^(100%). Anal. Calcd. for C_15_H_13_ClN_2_O_6_S_2_: C, 43.22; H, 3.14; N, 6.72; S, 15.38. Found: C, 43.25; H, 3.15; N, 6.70; S, 15.40.

*8-(2,5-dichlorophenyl)-2,3,4,5,6,8-hexahydrodithieno[3,2-*b*:2´,3´-*e*]pyridine-1,1,7,7-tetraoxide (Compond 3)*

Yield: 78%. m.p. 224-226 ^o^C. IR (ν, cm^-1^): 3332 (N-H), 1655 (C=O), 1284, 1125 (S=O). ^1^H-NMR (*δ*, DMSO-*d*_6_): 2.10-3.38 (8H; m; H^2,3,5,6^), 4.49 (H; s; H^8^), 5.86 (H; s; NH), 7.32 (1H; dd; J: 2.4 / 8.8 Hz; Ar-H^4^), 7.57 (1H; d; J: 8.8 Hz; Ar-H^3^), 8.14 (1H; d; J: 2.4 Hz; Ar-H^6^), ESI-MS (m/z): 428.96 [M+1+Na]^+^, 427.96 [M+Na]^+ ^(100%). Anal. Calcd. for C_15_H_13_Cl_2_NO_4_S_2_: C, 44.34; H, 3.23; N, 3.45; S, 15.78. Found: C, 44.37; H, 3.25; N, 3.45; S, 15.75.

*8-(2,3-dichlorophenyl)-2,3,4,5,6,8-hexahydrodithieno[3,2-*b*:2’,3’-*e*]pyridine-1,1,7,7-tetraoxide (Compond 4)*

Yield: 78%. m.p. 252-254 ^o^C. IR (ν, cm^-1^): 3339 (N-H), 1683 (C=O), 1304, 1132 (S=O). ^1^H-NMR (*δ*, DMSO-*d*_6_): 2.15-3.48 (8H; m; H^2,3,5,6^), 4.58 (H; s; H^8^), 7.29 (1H; t; J: 7.6 Hz; Ar-H^5^), 7.45 (1H; dd; J: 1.2 / 7.6 Hz; Ar-H^4^), 7.55 (1H; dd; J: 1.2 / 8 Hz; Ar-H^5^), 7.95 (H; s; NH). ESI-MS (m/z): 428.96 [M+1+Na]^+^, 427.96 [M+Na]^+ ^(100%). Anal. Calcd. for C_15_H_13_Cl_2_NO_4_S_2_: C, 44.34; H, 3.23; N, 3.45; S, 15.78. Found: C, 44.30; H, 3.24; N, 3.43; S, 15.72.

*8-(2-fluoro-3-chlorophenyl)-2,3,4,5,6,8-hexahydrodithieno[3,2-*b*:2´,3´-*e*]pyridine-1,1,7,7-tetraoxide (Compond 5)*

Yield: 75%. m.p. 218-220 ^o^C. IR (ν, cm^-1^): 3341 (N-H), 1675 (C=O), 1287, 1127 (S=O). ^1^H-NMR (*δ*, DMSO-*d*_6_): 2.18-3.42 (8H; m; H^2,3,5,6^), 4.50 (H; s; H^8^), 7.35 (1H; dd; J: 1.2 / 8.0 Hz; Ar-H^4^), 7.48 (1H; t; J: 8.0 Hz; Ar-H^5^), 7.60 (1H; dd; J: 1.2/ 8 Hz; Ar-H^6^), 7.87 (H; s; NH). ESI-MS (m/z): 412.99 [M+1+Na]^+^, 411.99 [M+Na]^+ ^(100%). Anal. Calcd. for C_15_H_13_ClFNO_4_S_2_: C, 46.21; H, 3.36; N, 3.59; S, 16.45. Found: C, 46.23; H, 3.34; N, 3.61; S, 16.42.

*8-(2-chloro-3-trifluoromethylphenyl)-2,3,4,5,6,8-hexahydrodithieno[3,2-*b*:2´,3´-*e*]pyridine-1,1,7,7-tetraoxide (Compond 6)*

Yield: 77%. m.p. 235-237 ^o^C. IR (ν, cm^-1^): 3338 (N-H), 1687 (C=O), 1315, 1136 (S=O). ^1^H-NMR (*δ*, DMSO-*d*_6_): 2.11-3.23 (8H; m; H^2,3,5,6^), 4.53 (H; s; H^8^), 7.25 (1H; t; J: 8.0 Hz; Ar-H^5^), 7.45 (1H; dd; J: 1.2 / 8.0 Hz; Ar-H^6^), 7.55 (1H; dd; J: 1.2/ 8 Hz; Ar-H^4^), 7.90 (H; s; NH). ESI-MS (m/z): 462.99 [M+1+Na]^+^, 461.99 [M+Na]^+ ^(100%). Anal. Calcd. for C_16_H_13_ClF_3_NO_4_S_2_: C, 43.69; H, 2.98; N, 3.18; S, 14.58. Found: C, 43.66; H, 2.99; N, 3.15; S, 14.61.

*7,7-Dimethyl-9-(2-chloro-5-nitrophenyl)-2,3,5,6,7,9-hexahydrothieno[3,2-b]quinolin-8(4H)-one, 1,1-dioxide (Compond 7)*

Yield: 68 %. m.p. 235-237 ^o^C. IR (ν, cm^-1^): 3354 (N-H), 1245, 1084 (S=O). ^1^H-NMR (*δ*, DMSO-*d*_6_): 0.86 (3H; s; 7-CH_3_), 1.01 (3H; s; 7-CH_3_), 1.72-3.36 (8H; m; H^2,3,5,6^), 4.89 (H; s; H^9^), 7.25 (1H; d; J: 9,2 Hz; Ar-H^3^), 7.82 (1H; dd; J: 2,4 / 9,2 Hz; Ar-H^4^), 7.95 (1H; d; J: 2,4 Hz; Ar-H^6^), 9.78 (H; s; NH). ESI-MS (m/z): 446.11 [M+1+Na]^+^, 445.11 [M+Na]^+ ^(100%). Anal. Calcd. for C_19_H_19_ClN_2_O_5_S: C, 53.97; H, 4.53; N, 6.62; S, 7.78. Found: C, 53.95; H, 4.55; N, 6.65; S, 7.80.

*7,7-Dimethyl-9-(2-nitro-5-chlorophenyl)-2,3,5,6,7,9-hexahydrothieno[3,2-b]quinolin-8(4H)-one, 1,1-dioxide (Compond 8)*

Yield: 68 %. m.p. 225-227 ^o^C. IR (ν, cm^-1^): 3365 (N-H), 1304, 1133 (S=O). ^1^H-NMR (*δ*, DMSO-*d*_6_): 0.83 (3H; s; 7-CH_3_), 0.94 (3H; s; 7-CH_3_), 1.53-3.38 (8H; m; H^2,3,5,6^), 5.12 (H; s; H^9^), 7.45 (1H; d; J: 8.8 Hz; Ar-H^3^), 7.88 (1H; d; J: 2.4 Hz; Ar-H^6^), 8.23 (1H; dd; J: 2.4 / 8.8 Hz; Ar-H^4^), 9.32 (H; s; NH). ESI-MS (m/z): 446.07 [M+1+Na]^+^, 445.07 [M+Na]^+ ^(100%). Anal. Calcd. for C_19_H_19_ClN_2_O_5_S: C, 53.97; H, 4.53; N, 6.62; S, 7.78. Found: C, 53.94; H, 4.52; N, 6.62; s, 7.76.

*7,7-Dimethyl-9-(2,5-dichlorophenyl)-2,3,5,6,7,9-hexahydrothieno[3,2-b]quinolin-8(4H)-one, 1,1-dioxide (Compond 9)*

Yield: 76 %. m.p. 252-254 ^o^C. IR (ν, cm^-1^): 3341 (N-H), 1288, 1093 (S=O). ^1^H-NMR (*δ*, DMSO-*d*_6_): 0.90 (3H; s; 7-CH_3_), 1.00 (3H; s; 7-CH_3_), 1.68-3.42 (8H; m; H^2,3,5,6^), 5.21 (H; s; H^9^), 7.05 (1H; d; J: 7.6 Hz; Ar-H^3^), 7.28 (1H; d; J: 2,4 Hz; Ar-H^6^), 7.61 (1H; dd; J: 2,4 / 8,4 Hz; Ar-H^4^), 9.59 (H; s; NH). ESI-MS (m/z): 435.04 [M+1+Na]^+^, 434.04 [M+Na]^+ ^(100%). Anal. Calcd. for C_19_H_19_Cl_2_NO_3_S: C, 55.35; H, 4.64; N, 3.40; S, 7.78. Found: C, 55.38; H, 4.64; N, 3.38; S, 7.75.

*7,7-Dimethyl-9-(2,3-dichlorophenyl)-2,3,5,6,7,9-hexahydrothieno[3,2-b]quinolin-8(4H)-one, 1,1-dioxide (Compound 10)*

Yield: 72 %. m.p. 265-267 ^o^C. IR (ν, cm^-1^): 3346 (N-H), 1305, 1125 (S=O). ^1^H-NMR (*δ*, DMSO-*d*_6_): 0.84 (3H; s; 7-CH_3_), 0.96 (3H; s; 7-CH_3_), 1.75-3.44 (8H; m; H^2,3,5,6^), 5.30 (H; s; H^9^), 7.17 (1H; dd; J: 1.6 / 8 Hz; Ar-H^4^), 7.22 (1H; t; J: 8 Hz; Ar-H^5^), 7.38 (1H; dd; J: 1.6 / 8 Hz; Ar-H^6^), 9.76 (H; s; NH). ESI-MS (m/z): 435.04 [M+1+Na]^+^, 434.04 [M+Na]^+ ^(100%). Anal. Calcd. for C_19_H_19_Cl_2_NO_3_S: C, 55.35; H, 4.64; N, 3.40; S, 7.78. Found: C, 55.31; H, 4.66; N, 3.43; s, 7.72.

*7,7-Dimethyl-9-(2-fluoro-3-chlorophenyl)-2,3,5,6,7,9-hexahydrothieno[3,2-b]quinolin-8(4H)-one, 1,1-dioxide (Compound 11)*

Yield: 70 %. m.p. 216-218 ^o^C. IR (ν, cm^-1^): 3365 (N-H), 1299, 1075 (S=O). ^1^H-NMR (*δ*, DMSO-*d*_6_): 0.82 (3H; s; 7-CH_3_), 0.91 (3H; s; 7-CH_3_), 1.95-3.38 (8H; m; H^2,3,5,6^), 5.32 (H; s; H^9^), 7.20 (1H; dd; J: 2.0 / 8.4 Hz; Ar-H^4^), 7.34 (1H; t; J: 8.4 Hz; Ar-H^5^), 7.42 (1H; dd; J: 2.0 / 8.4 Hz; Ar-H^6^), 9.85 (H; s; NH). ESI-MS (m/z): 389.07 [M+1+Na]^+^, 388.07 [M+Na]^+ ^(100%). Anal. Calcd. for C_19_H_19_ClFNO_3_S: C, 57.65; H, 4.84; N, 3.54; S, 8.10. Found: C, 57.69; H, 4.86; N, 3.53; S, 8.12.

*7,7-Dimethyl-9-(2-chloro-3-trifluoromethylphenyl)-2,3,5,6,7,9-hexahydrothieno[3,2-b]quinolin-8(4H)-one, 1,1-dioxide (Compound 12)*

Yield: 82%. m.p. 241-243 ^o^C. IR (ν, cm^-1^): 3346 (N-H), 1355, 1129 (S=O). ^1^H-NMR (*δ*, DMSO-*d*_6_): 0.80 (3H; s; 7-CH_3_), 0.89 (3H; s; 7-CH_3_),1.62-3.14 (8H; m; H^2,3,5,6^), 5.22 (H; s; H^9^), 7.34 (1H; dd; J: 1.6 / 7.6 Hz; Ar-H^4^), 7.52 (1H; t; J: 7.6 Hz; Ar-H^5^), 7.67 (1H; dd; J: 1.6 / 7.6 Hz; Ar-H^6^), 9.75 (N-H). ESI-MS (m/z): 469.05 [M+1+Na]^+^, 468.05 [M+Na]^+ ^(100%). Anal. Calcd. for C_20_H_19_ClF_3_NO_3_S: C, 53.88; H, 4.30; N, 3.14; S, 7.19. Found: C, 53.90; H, 4.31; N, 3.16; S, 7.21.

*Pharmacology*

The study protocol was approved by Hacettepe University Animal Ethics Committee (2012-54/02)

Male Sprague-Dawley rats (250-350 g) were used in the present study. The rats were euthanized by CO_2 _inhalation and decapitation. The superior mesenteric arteries and the urinary bladders were isolated and placed in a Krebs-Henseleit solution. The composition of the Krebs-Henseleit solution was (in mM ): NaCI, 113; KCI, 4.7; MgSO_4_, 1.2; CaCI_2_, 2.5; KH_2_PO_4_, 1.2; NaHCO_3_, 25.0; and glucose, 11.6. The solution was gassed with a mixture of 95% O_2_- 5% CO_2 _and maintained at 37 °C and pH 7.4. Pinacidil monohydrate, glibenclamide, tetraethylammonium chloride were purchased from Sigma (St. Louis. MO).

*Preparation of the tissues*

*Superior mesenteric artery: *The superior mesenteric artery was cut into rings of 2-3 mm length. No attempt was made to remove the endothelium. The rings were suspended between two stainless-steel hooks under a resting tension of 1g in 5 mL organ baths filled with Krebs-Henseleit solution. 

*Urinary bladder*: 

Urinary bladder strips about 1.5 mm wide and 10 mm long were prepared and were mounted under a resting tension of 1 g in 5 mL organ-baths filled with Krebs-Henseleit solution. Urothelium was not removed from the bladder strips. 

Isometric changes in the tension were measured and recorded with an isometric force transducer, Biopac data acquisition system MP150 and Acknowledge 4.2 software**.** Tissues were equilibrated for 1.5 h and washed by Krebs-Henseleit solution every 15 min before each experimental procedure. 

*Experimental procedure*

*Superior mesenteric artery:*

Three-ring preparations were obtained from each mesenteric artery, and the rings were used to elicit the relaxation response to each test compound either in the absence or in the presence of the antagonists; ATP-sensitive potassium channel blocker glibenclamide (10 µM) or calcium-activated potassium channel blocker tetraethylammonium (TEA) (1000 µM).

At the beginning of each experiment, after priming with KCI (60 mM) and one hour washout period, mesenteric artery rings were contracted to approximately 80% of the maximum contraction to phenylephrine (1 µM). When the contraction had plateaued, the relaxation response to cumulative concentrations of the test compound (10^-8^-10^-4^ M) was determined. The antagonists used were incubated for 30-min before obtaining the relaxation response to the test compound. Concentration-response curve to each compound was obtained in individual preparations. 

**Figure 2:**
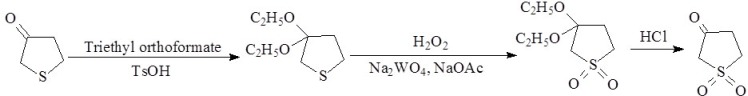
Synthesis of tetrahydrothiophene-3-one-1,1-dioxide

**Figure 3:**
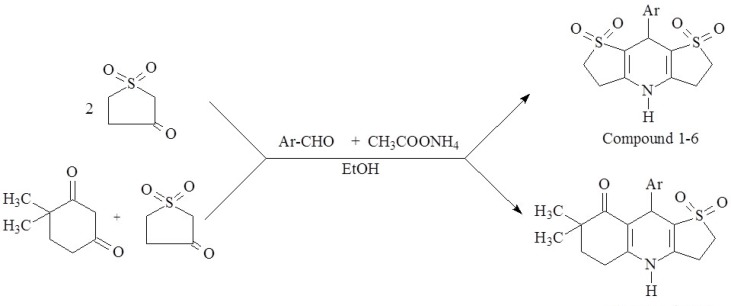
Synthesis of compound 1-12

**Figure 4:**
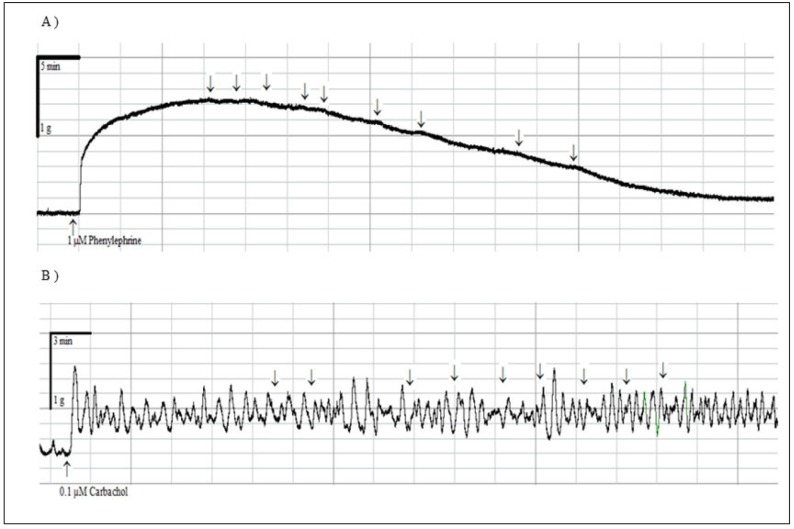
Representative traces showing concentration dependent relaxant effect of compound 9 (10^-8^-10^-4^) in precontracted rat mesenteric artery rings (A) and precontracted rat urinary bladder strips (B

**Figure 5:**
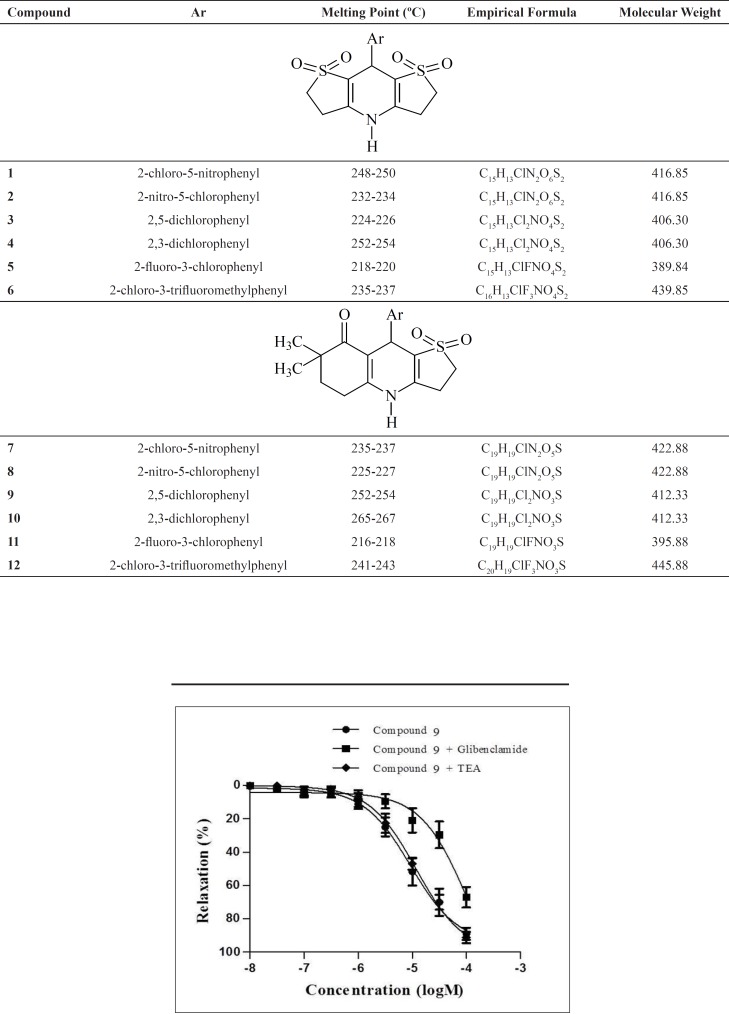
Concentration-response curves for the relaxant effect of compound 9 in precontracted rat mesenteric artery rings in the absence or presence of glibenclamide or TEA

**Figure 6:**
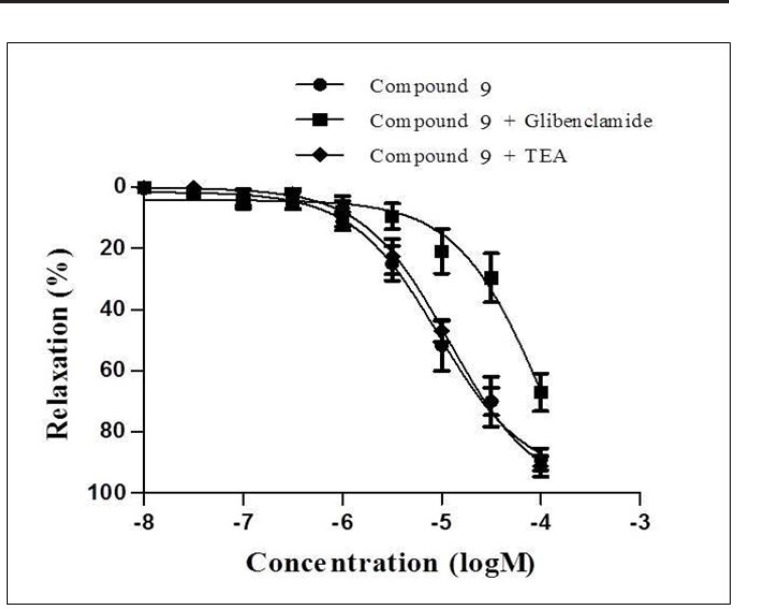
Binding conformation of compound 9 and the space occupied in the 1BL8 binding pocket (A) and Color-coded pharmacophore features and interactions of compound 9: hydrophobic interaction (yellow sphere), hydrogen bond acceptor (red vector), hydrogen bond donor (green vector

**Table 1:** Structural data of the synthesized compounds

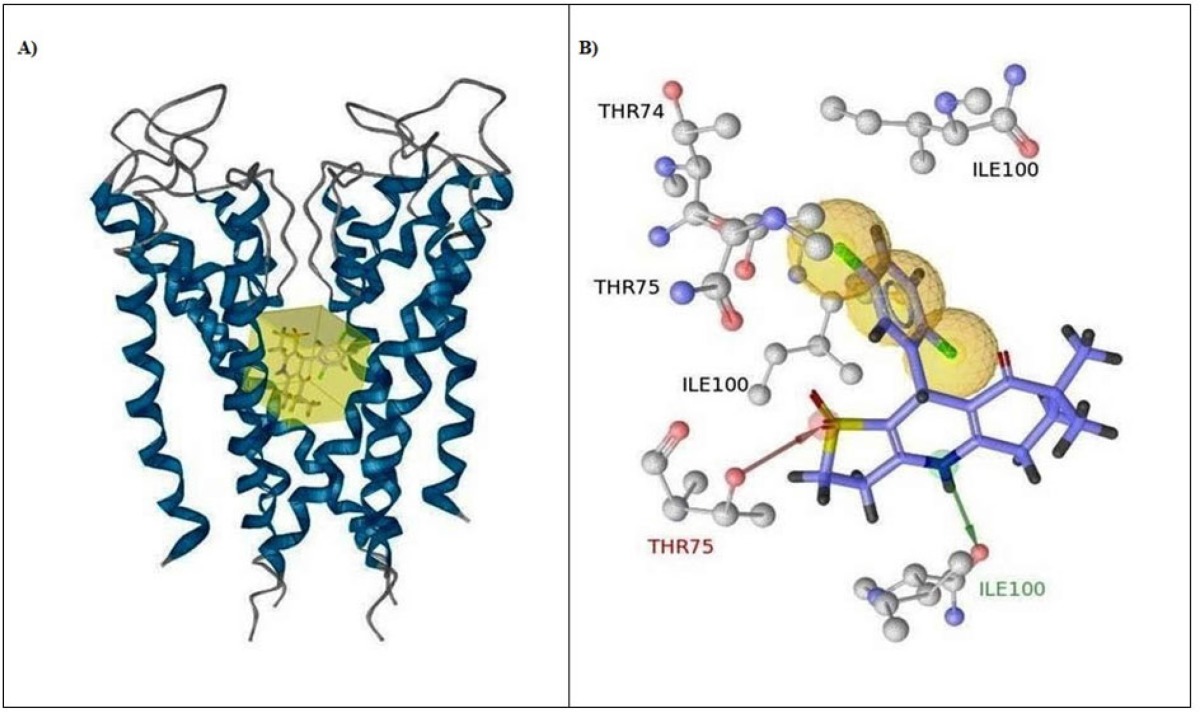

**Table 2:** E_max_ and pD_2_ values of the relaxation responses of pinacidil and the test compounds 1-12 in isolated rat mesenteric arteries precontracted with 1 µM phenylephrine in the absence or presence of glibenclamide or TEA

**Compound**	**E**_max_			**pD**_2_		
	**Control**	**Glibenclamide**	**TEA**	**Control**	**Glibenclamide**	**TEA**
Pinacidil	101.54 ± 0.96	93.25 ± 5.42		6.18 ± 0.11	4.64 ± 0.34^a^	
1	No effect	-	-	-	-	-
2	No effect	-	-	-	-	-
3	13.85 ± 3.23*	-	-	3.50 ± 1.58*	-	-
4	38.32 ± 6.75*	-	-	1.38 ± 0.10*	-	-
5	22.40 ± 2.91*	-	-	1.83 ± 1.18*	-	-
6	23.07 ± 8.11*	-	-	1.41 ± 1.20*	-	-
7	30.46 ± 6.52*	-	-	6.22 ± 0.46	-	-
8	53.05 ± 2,52*	-	-	7.87 ± 1,23	-	-
9	89.01 ± 3.60*	67.12 ± 6.21^a^	91.31 ± 3.42	4.99 ± 0.16*	2.62 ± 1.12^a^	4.93 ± 0.08
10	72.82 ± 2.78 *	39,79 ± 7,71^a^	70.12 ± 2,34	5.14 ± 0.08*	4.41 ± 0.29^a^	4.98 ± 0.02
11	77.62 ± 4.84*	49.88 ± 6.17^a^	73.82 ± 6.41	4.69 ± 0.13*	3.51 ± 0.77^a^	4.45 ± 0.19
12	62.73 ± 6.36*	35.70 ± 8.14^a^	63.49 ± 7.13	5.07 ± 0.20*	3.01 ± 1.25^a^	3.75 ± 1.31

The relaxation response to compounds are given as the percentage of phenylephrine (10^-6 ^M) induced precontraction. The maximum effects of compounds (E_max_) and the negative logarithm values of concentration for half maximal effect (pD_2_) are presented as mean value ± S.E.M (n=4-5).

* Significantly different from pinacidil *p*0.05.

a Significantly different from E_max, _pD_2 _values of test compounds and pinacidil in the absence of glibenclamide *p*0.05.

**Table 3:** Computer aided ADME study of compound 9-12.

**Compound**	**Slog p** ^a^	**ASA_P** ^b^	**logS** ^c^	**Solubility Level** ^d^	**HIA** ^e^	**CYP2D6** ^f^
**9**	4.35	92.397	-4.887	2	0	Non inhibitor
**10**	4.35	88.932	-4.887	2	0	Non inhibitor
**11**	3.83	92.932	-4.448	2	0	Non inhibitor
**12**	4.51	181.392	-5.210	2	0	Non inhibitor

a Lipophilicity descriptor

**Table 4:** Lipinski parameters of compound 9-12.

**Compound**	**Number of HBA**	**Number of HBD**	**Slog p**	**Molecular Weight **
**9**	3	1	4.35	412.33
**10**	3	1	4.35	412.33
**11**	3	1	3.83	395.88
**12**	3	1	4.51	445.88

*Urinary bladder*: 

Two-longitudinal strips were prepared from each urinary bladder and the concentration-dependent relaxation response to the test compound was obtained either in the absence or in the presence of 10 µM glibenclamide. 

The bladder strips were precontracted with 0.1 µM carbachol to obtain submaximal contraction, and after the contractile response reached plateau, the increasing concentrations of test compound (10^-8^-10^-4^ M) was added to the organ bath in a cumulative manner. Concentration-response curve to each test compound was obtained in individual preparations. 

*Statistical analysis*

The relaxation responses to the test compounds and pinacidil were expressed as the percentage of phenylephrine (for mesenteric artery rings) and carbachol (for urinary bladder strips)-induced precontractions. 

The maximum response (E_max_) as the efficacy, and the pD_2_ values ( the negative logarithm of the concentration of the compound producing 50 % (EC_50_) of the maximum relaxation) as the potency index were calculated from each concentration-response curve.

Stock solutions of the compounds were dissolved in dimethyl sulphoxide (DMSO). Final concentration of DMSO in the bath did not exceed 0.05%. Further dilutions were made in distilled water. 

Data are represented as mean±standard error of mean (SEM). Statistical analysis was done by Student’s *t* test and analysis of variance (ANOVA) followed by the Bonferroni test by using GraphPad Prism5 software. P<0.05 was considered as significant. 

*Computational methodology*

*Molecular Docking Studies*

*Ligand Preparation*

The chemical formulas of the compounds were drawn in Chembiodraw Ultra 12.0 and saved as Simplified Molecule Input Entry System (SMILES) file. The file was transfered to LigandScout 3.1. (30) in order to prepare the appropriate file needed for the docking study. For this purpose, the structures were geometrically optimized and energy minimized to 3D structure using the MMFF94x force field in LigandScout 3.1.

*Protein preparation*

The reported X-ray crystal structure of potassium channel receptor (KcsA) of Streptomyces lividans (PDB code: 1BL8), an integral membrane protein with sequence similarity to all known K^+^ channels, was obtained from the Protein Data Bank of Brookhaven (PDB, www.rcsb.org/pdb) (31). The structure of the protein was transferred to GOLD (Genetically Optimized Ligand Docking) and prepared by removing water molecules and metal ions and adding hydrogen atoms before docking.

*Docking procedure*

The binding region was identified by the help of recent studies about the same protein (9, 31, 32). Docking runs were performed using standard default parameters. Ten docking poses were obtained for each ligand and the scoring function GoldScore implemented in GOLD was used to rank the docking poses of the molecules. LigandScout was used for the further analysis of the conformation of molecules based on the best fitness scores. 

*ADME and drug likeness prediction*

The ADME of compound 9-12 was predicted via a theoretical study that performed by means of MOE (Chemical Computing Group) and PreADMET (http://preadmet.bmdrc.org/). logP and ASA_P descriptors were calculated to evaluate the lipophilicity and polar surface area. Also, solubility and CYP2D inhibition levels were predicted. Lipinski’s “rule of five” has been also calculated as an attempt to predict the drug likeness of the active compounds by the help of ligand properties tool implemented in MOE.

## Results and Discussion

*Chemistry*

The synthetic route used to synthesize tetrahydrothiophene-3-one-1,1-dioxide and the test compound 1-12 have been depicted in Figure 2 and Figure 3. respectively. The ketone group of tetrahydrothiophen-3-one was protected by converting it temporarily to a ketal by reacting the starting compound with triethyl orthoformate and catalytic amount of acid. Hydrogen peroxide was used to oxidize the thioeter group and finally the ketal group was converted back into a ketone through acid-catalyzed hydrolysis to form tetrahydrothiophene-3-one-1,1-dioxide.

The desirable target compounds were prepared by one-pot synthesis of tetrahydrothiophene-3-one-1,1-dioxide (and) 4,4-dimethyl-1,3-cyclohexanedione, appropriate disubstituted benzaldehyde and ammonium acetate under microwave irradiation in ethanol, which was classified as an excellent microwave-absorbing solvent (25, 33).

The appearance of the products was monitored by TLC and the reaction time was determined as 5 min., which is quite a short time for the preparation of similar tricyclic 1,4-DHP compounds (23, 34)

Structures and chemical characteristics of the synthesized compounds are presented in Table 1. 

The structures of the synthesized compounds were elucidated by spectral methods (IR, ^1^H-NMR and mass spectra) and confirmed by elemental analysis. In the IR spectra, characteristic N-H, S=O and C=O (compound 7-12) stretching bonds were observed.

The mass spectra of the compounds were recorded via the electrospray ionization technique. The quasimolecular ions created by the addition of a hydrogen cation [M + H]^+^ and of sodium ion [M + Na]^+^ were observed in the spectra of all compounds. Cleavage of the substituted phenyl ring from the parent molecule is the next most observed fragmentations.

Elemental analysis results were within ± 0.4% of the theoretical values for all compounds.

*Pharmacology*

Potassium channel opening activities of the compounds 1-12 and pinacidil were determined on isolated rat mesenteric artery ring and isolated rat urinary bladder strip preparations. The mechanism of the relaxation responses of the selected compounds were investigated in the presence of K_ATP_ channel antagonist glibenclamide and K_Ca_ channel antagonist tetraethylammonium (TEA). The maximum relaxant effects (E_max_) and pD_2_ values [the negative logarithm of the concentration for the half-maximal response (EC_50_)] of compound 1-12 and pinacidil in isolated rat mesenteric arteries in the absence and presence of glibenclamide or TEA are given in Table 2.

Compound 3-12 (10^-8^-10^-4^ M) and pinacidil induced concentration-dependent relaxation responses in precontracted mesenteric artery rings; although compound 1 and 2 did not cause any relaxation response and even did not alter the phenylephrine-induced precontraction level of mesenteric arteries. 

None of the compounds 1-12 exerted relaxant effect in precontracted rat urinary bladder strips (Data not shown). These obtained results suggested that the compounds produced mesenteric artery-selective relaxant responses. The representative traces of compound 9 with the highest efficacy on mesenteric artery rings (A) and precontracted rat urinary bladder strips (B) are given in Figure 4.

Subsequently, compound 9-12, which exhibited more than 60% inhibition on mesenteric arteries, were chosen for their higher potencies in order to evaluate the underlying mechanism of the relaxant effect of these compounds. The relaxation responses of these compounds were elicited in the presence of K_ATP_ channel antagonist glibenclamide and K_Ca_ channel antagonist TEA. Concentration- response curves of compounds 9-12 were shifted to the right with glibenclamide. Moreover, the relaxation responses of these compounds were not changed by K_Ca_ channel antagonist TEA. These data suggested that the relaxant effects of the compound 9-12 were mediated through 

K_ATP _channels and K_Ca_ channels did not play role in the relaxation effects of these compounds. The concentration-response curves of compound 9 in precontracted rat mesenteric artery rings in the absence or presence of glibenclamide or TEA are given Figure 5.

*Molecular Docking*

Molecular docking studies of the Compounds 1-12 in the active site of potassium channel (PDB code:1BL8) were performed to get further information about the type of interactions between the compounds and the active site amino acids to rationalize the obtained biological results. Binding conformation of the most active compound (Compound 9) with the space occupied (yellow box around the ligand) in the 1BL8 binding pocket (A) and the pharmacophore features and 3D interactions of the Compound 9 with the binding site of the protein (B) have been showed in Figure 6.

The unsubstituted nitrogen atom on the 1,4-DHP ring was important for the hydrogen bonding with the carbonyl group of ILE100 and one of the sulfonyl oxygens was placed close to the OH group of THR75 also for the formation of hydrogen bond. These two key hydrogen bonds, donor and acceptor respectively, with ILE100 and THR75 stabilized the compound in the center of the cavity. The phenyl ring is positioned in the hydrophobic binding pocket surrounded by THR74, THR75, ILE100 and PHE103. When the obtained findings were analyzed with respect to the substitution of the phenyl ring, it was observed that compared to chlorine atoms; relatively bulkier substituents like nitro and trifluoromethyl prevented the compounds to orient in the cavity. 2,5-dichloro substitution on the phenyl ring led to more hydrophobic interactions with the protein.

*ADME and drug likeness prediction*

Theoretical calculations were carried out to predict the ADME of compound 9-12 (Table 3). Active compounds were expected to have low solubility. Whilst, all the examined derivatives were seemed to possess good absorption levels and predicted to be CYP2D non-inhibitors. Notably, the compounds passed the Lipinski›s rule of five (Log p < 5, MW < 500, number of hydrogen bond donors (HBD) < 5 and number of hydrogen bond acceptors (HBA) < 10). (Table 4)

## Conclusion

We reported herein a very rapid and convenient microwave-assisted method for the preparation of novel tricyclic 1,4-DHPs. This method also offers a reduction of solvent use and simplification of the work-up procedures. The potassium channel opening effects of the compounds were determined on rat mesenteric arteries and urinary bladders. The obtained results indicated that the compound 3-12 produced mesenteric artery-selective relaxant properties over bladder and replacement of the second tetrahydrothiophene ring with dimethyl-substituted cyclohexane ring led to more active compounds. The activity of the compound 9-12 were found to be mediated through K_ATP _channels and K_Ca_ channels did not play role in the relaxation effects of these compounds. The potassium channel opening ability of these effective agents needs further investigations.

Docking studies revealed that the nitrogen atom of the 1,4-DHP ring must be unsubstituted for the formation of hydrogen bond. Small substituents at 2 and 5-position of the phenyl ring could be preferred for the potassium channel opening activity. 

## Conflict of interest

All authors of the article declare no conflict of interest.
